# MDN-0170, a New Napyradiomycin from *Streptomyces* sp*.* Strain CA-271078

**DOI:** 10.3390/md14100188

**Published:** 2016-10-18

**Authors:** Rodney Lacret, Ignacio Pérez-Victoria, Daniel Oves-Costales, Mercedes de la Cruz, Elizabeth Domingo, Jesús Martín, Caridad Díaz, Francisca Vicente, Olga Genilloud, Fernando Reyes

**Affiliations:** Fundación MEDINA, Centro de Excelencia en Investigación de Medicamentos Innovadores en Andalucía, Avda. del Conocimiento 34, 18016 Armilla (Granada), Spain; ignacio.perez-victoria@medinaandalucia.es (I.P.-V.); daniel.oves@medinaandalucia.es (D.O.-C.); mercedes.delacruz@medinaandalucia.es (M.d.l.C.); elidc85@hotmail.com (E.D.); jesus.martin@medinaandalucia.es (J.M.); caridad.diaz@medinaandalucia.es (C.D.); francisca.vicente@medinaandalucia.es (F.V.); olga.genilloud@medinaandalucia.es (O.G.)

**Keywords:** *Streptomyces*, napyradiomycin, structural elucidation, antimicrobial activity

## Abstract

A new napyradiomycin, MDN-0170 (**1**), was isolated from the culture broth of the marine-derived actinomycete strain CA-271078, together with three known related compounds identified as 4-dehydro-4a-dechloronapyradiomycin A1 (**2**), napyradiomycin A1 (**3**) and 3-chloro-6,8-dihydroxy-8-α-lapachone (**4**). The structure of the new compound was determined using a combination of spectroscopic techniques, including 1D and 2D NMR and electrospray-time of flight mass spectrometry (ESI-TOF MS). The relative configuration of compound **1,** which contains two independent stereoclusters, has been established by molecular modelling in combination with nOe and coupling constant analyses. Biosynthetic arguments also allowed us to propose its absolute stereochemistry. The antimicrobial properties of the compounds isolated were evaluated against methicillin-resistant *Staphylococcus aureus* (MRSA), *Escherichia coli*, *Aspergillus fumigatus*, and *Candida albicans*. The potent bioactivity previously reported for compounds **2** and **3** against methicillin-sensitive *S. aureus* has been extended to methicillin-resistant strains in this report.

## 1. Introduction

The napyradiomycins (NPDs) constitute an interesting family of halogenated natural compounds mainly produced by bacteria of the family *Streptomycetaceae*, which were first discovered from cultures of the actinomycete *Chainia rubra* [1,2], later reclassified as *Streptomyces ruber* [3]. This class of secondary metabolites consists of a naphthoquinone core, a prenyl unit attached at C-4a, a monoterpenoid substituent at C-10a, and some congeners have a methyl group at C-7 [1,4,5,6]. At present, nearly 47 different NPDs have been described [1,2,4,5,6,7,8,9,10,11,12]. Compounds belonging to this structural class possess significant antibacterial activity against pathogenic bacterial strains such as methicillin-resistant *Staphylococcus aureus* (MRSA) and inhibit the growth of several tumor cell lines [13,14,15]. In the course of our continuous search for new bioactive natural products from marine actinomycetes, over 400 marine-derived strains were grown in carefully selected media and their fermentations extracts were assayed against clinically relevant pathogenic microbial strains [16]. 

Growth inhibition of MRSA was observed in the acetone crude extract from fermentation broths of strain CA-271078, which upon 16S rRNA sequencing was found to be closely related to *Streptomyces aculeolatus* NBRC 14824(T). A bioassay-guided fractionation of the ethyl acetate extract of this microorganism and a dereplication by LC/MS of the bioactive fractions was carried out in order to isolate and identify the new chemical constituents that were responsible for the activities observed. Herein we report the isolation of MDN-0170 (**1**), a new napyradiomycin alongside three related known compounds (**2**–**4**). The structural elucidation of MDN-0170 was accomplished using a combination of spectroscopic techniques, including HRMS and extensive 1D and 2D NMR analyses in combination with molecular modelling.

## 2. Results and Discussion

### 2.1. Isolation and Taxonomy of the Producing Microorganism

**Figure 1:**
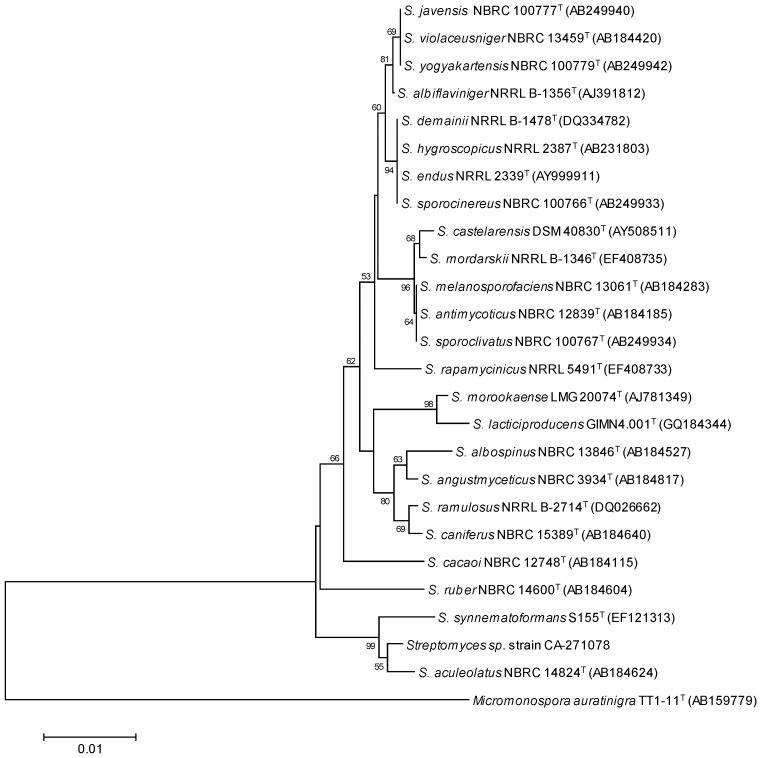
Neighbor-joining (NJ) tree built with MEGA 6.06 based on nearly-complete 16S rRNA gene sequences of CA-271078 and the closest type strains of the genus *Streptomyces*. *Micromonospora auratinigra* TT1-11(T) was employed as out-group. The numbers at the nodes indicate bootstrap support (%) based on NJ analysis of 1000 replicates; only values higher that 50% are shown. The scale bar indicates 0.01 substitutions per site.

The producing strain, CA-271078, was isolated from an ascidian collected at the sea shore in Baía Ana Chaves, Sao Tome (Sao Tome and Principe). A BLASTN search employing the PCR-amplified 16S rRNA sequence (1359 bp) indicated that the strain was related to *Streptomyces aculeolatus* NBRC 14824(T) (99.34% similarity) [17]. A phylogenetic tree was constructed using the neighbor-joining method corrected with the Jukes-Cantor algorithm [18,19] (Figure 1) showing the relatedness with *Streptomyces aculeolatus* NBRC 14824(T) (99.34% similarity) and *Streptomyces synnematoformans* S155(T) (98.73% similarity). The remaining closest members of the genus *Streptomyces* exhibited sequence similarities below 98%. These data strongly indicate that strain CA-271078 is a member of the genus *Streptomyces*.

### 2.2. Extraction, Dereplication and Bioassay-Guided Isolation

The producing strain CA-271078 was fermented at 28 °C in 1 L of R358 medium for six days. Extraction with an equal volume of acetone and evaporation of the organic solvent afforded an acetone crude extract, which was subsequently subjected to liquid-liquid extraction with ethyl acetate (EtOAc). This organic extract showed antibacterial activity against MRSA. LC-UV-MS analysis of the ethyl acetate extract revealed the presence of some compounds that were not included in our in-house microbial natural products library [20] nor in the Chapman & Hall dictionary of Natural Products [21].

**Figure 2:**
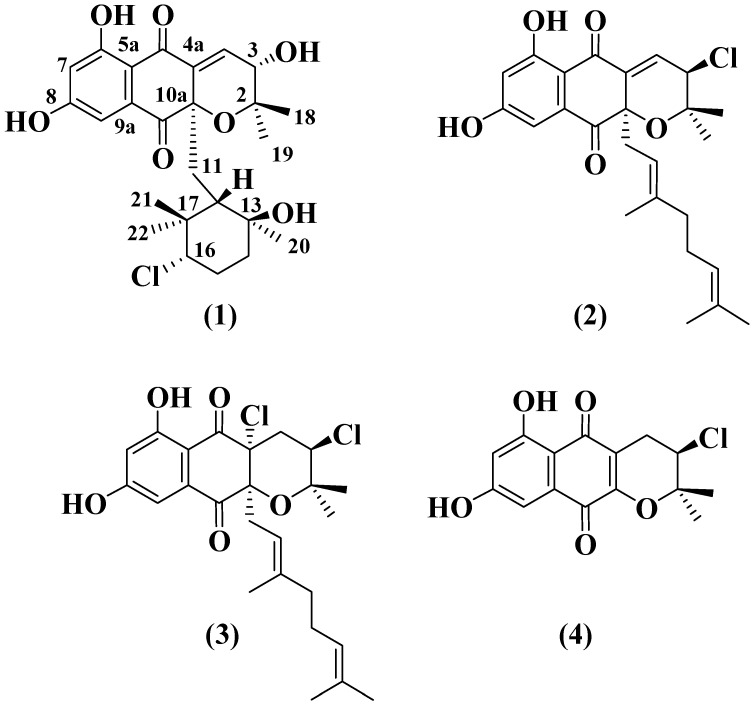
Compounds isolated from culture broths of *Streptomyces* sp. CA-271078.

The ethyl acetate extract was chromatographed on Sephadex LH-20 using dichloromethane/methanol to afford ten fractions: A–J. Fractions C, D, F, G, and H were the most active in a test against MRSA. LC-DAD-HRMS analysis allowed us to detect possible milbemycin-like antibiotics in fractions C and D [4,22]. This analysis also showed that fractions F, G, and H contained possible known and bioactive NDPs bearing chlorine (according to their isotopic pattern), such as napyradiomycin A1 and 4-dehydro-4a-dechloronapyradiomycin A1 [8,12]. Additionally, these fractions also contained minor amounts of related NPDs whose molecular formulae suggested their novelty as natural products since they were not found in the Dictionary of Natural Products [21]. According to the abundance, chemical profile and our interest in new NPDs, we decided to continue the bioassay-guided isolation with fractions F, G, and H. Further chromatographic separation of bioactive fractions (F, G, and H) on Sephadex LH-20 and on reversed phase C8 HPLC using a gradient of CH_3_CN/H_2_O, allowed us to isolate four compounds (Figure 2). MDN-0170 (**1**) was identified as a new compound on the basis of ESI-TOF and NMR analysis. The spectroscopic data of **2**–**4** were identical to those previously reported for 4-dehydro-4a-dechloronapyradiomycin A1 (**2**) [12], napyradiomycin A1 (**3**) [1,12] and 3-chloro-6,8-dihydroxy-8-α-lapachone (**4**) [12].

### 2.3. Structural Determination of MDN-0170 *(**1**)*

Compound **1** was isolated as a yellow oil. Its molecular formula was determined as C_25_H_31_ClO_7_ on the basis of ESI-TOF measurements (*m/z* 479.1818 [M + H]^+^, calcd. for C_25_H_32_^35^ClO_7_^+^, 479.1831) accounting for ten degrees of unsaturation. The UV absorption pattern, with maxima at 258, 310 and 362 nm of **1** suggested that this compound possessed the dihydronaphthoquinone moiety typically observed in NPDs [6,8,12]. 

The ^1^H NMR spectrum of **1** (Table 1 and Figure S2) displayed two aromatic protons at δ 6.60 (1H, d, 2.0, H-7) and 6.96 (1H, d, 2.0, H-6), one downfield olefinic proton at δ 7.10 (1H, d, 6.9, H-3) that suggested the presence of a trisubstituted double bond attached to an electron withdrawing group, and two methine protons at δ 3.70 (1H, dd, 12.2, 4.0, H-16) and 3.88 (1H, d, 6.8, H-3). On the other hand, five methyl groups in the aliphatic region at 0.56 (3H, s, H-21), 0.71 (3H, s, H-22), 1.04 (3H, s, H-18), 1.17 (3H, s, H-20), and 1.44 (3H, s, H-19) could also be distinguished. The ^13^C NMR spectrum exhibited 25 signals: 5 methyl, 3 methylene, 6 methine, and 11 quaternary carbons, according to an heteronuclear single quantum coherence spectroscopy (HSQC) experiment (multiplicity edited). Eight of these quaternary carbons were two carbonyl groups (δ 190.6 and 196.3), two phenolic hydroxyl groups (δ 167.3 and 166.3), and four sp^3^ carbons (δ 41.8, 72.1, 78.8, and 83.4). The NMR data confirmed that compound **1** was structurally related to dihydronaphthoquinones with two terpenoid substituents (NPDs B series) [8,12].

**Figure 3:**
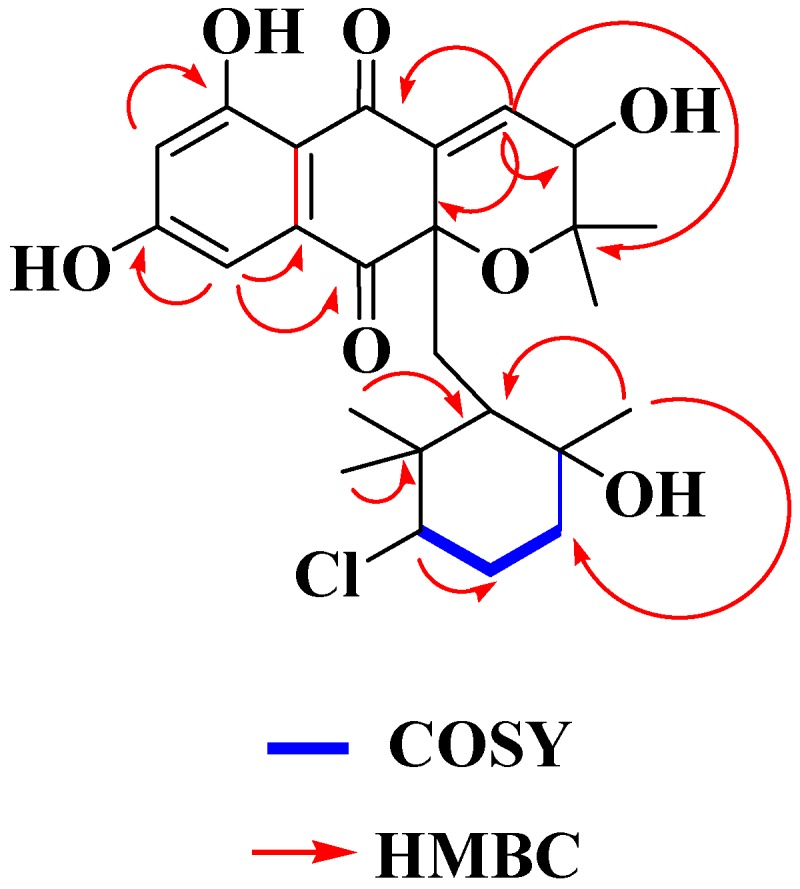
Key COSY and HMBC correlations observed in the spectra of compound **1**.

The COSY spectrum of **1** showed the presence of the fragment –CH_2_–CH_2_–CHX– (C-14 to C-16). Thus, the methylene that resonated at δ 1.82 (H-15 ax) and 1.91 (H-15 eq) showed a coupling with the signals at δ 3.70 (H-16), 1.55 (H-14a), and 1.77 (H-14b). The signal at δ 3.88 (1H, d, 6.9) was assigned to H-3 whose carbon signal appears at δ 67.0 and suggests that an oxygenated function is located at this position. All these proposals were further supported by the similarity of the spectroscopic data of **1** with those reported for other analogous compounds [5,8]. Correlations in the HMBC experiment (Figure 3) between the olefinic proton at δ_H_ 7.10 and the signals at δ_C_ 78.8 (C-2), 67.0 (C-3), 140.2 (C-4a), 190.6 (C-5), and 83.4 (C-10a) confirmed the position of the trisusbtituted double bond at Δ^4^ and the hydroxyl group at C-3. This NMR analysis thus allowed establishing the connectivity of Compound **1** as 3-hydroxy-10a-(3-chloro-6-hydroxy-2,2,6-trimethylcyclohexylmethyl)-6,8-dihydroxy-2,2-dimethyl-3,10a-dihydro-2H-benzo[*g*]chromene-5,10-dione, which was given the name MDN-0170 according to our proprietary compound library.

**Figure 4:**
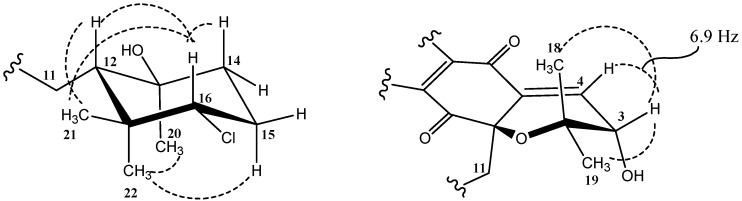
Key NOESY correlations (dashed lines) and coupling constant which determine the relative configuration of each stereocluster in MDN-0170 (**1**).

**Figure 5:**
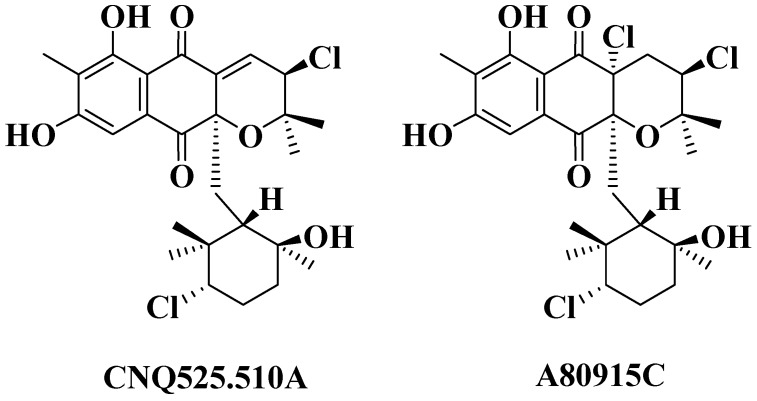
Structures of CNQ525.510A and A80915C.

The relative stereochemistry of MDN-0170 (**1**) was determined by analysis of key NOESY correlations (Figure 4) and the coupling constants observed in its ^1^H NMR spectrum in combination with molecular modelling. The biogenetic origin described for the napyradiomycin B series was also taken into account [5,23]. The cyclohexane ring displays the expected chair conformation in which H-16 is located in axial disposition since it appears as a double doublet with large (12.2 Hz) axial-axial and medium (4.0 Hz) axial-equatorial coupling constants to each of its vicinal methylene protons. On the basis of the strong NOESY correlation observed between H-16 and H-12, their 1-3 diaxial relationship on the same face of the ring was also clear. Additionally, the C-21 methyl group showed key NOESY correlations with both H-16 and H-12, indicating its equatorial display sharing the same face of the cyclohexane ring. On the other hand, the strong NOESY correlation between the C-20 and C-22 methyl groups indicated that they are axially displayed on the same face of the ring (opposite to the face where H-12 is located). The relative configuration on the cyclohexane ring was, therefore, identical to that reported for related compounds [2,4,5,6,24]. Thus, it is expected that the absolute configuration of the chiral centers on this cyclohexane ring is also the same since MDN-0170 (**1**) and the previously mentioned compounds must share a common biosynthetic route [21]. This same biogenetic argument prompted us to assume that the relative stereochemical relationship between C-10a and C-12, which connects the stereochemistry of the two independent stereoclusters (cyclohexane ring and dihydropyran ring), must be the same as that found for CNQ525.510A [5,6] (Figure 5).

**Figure 6:**
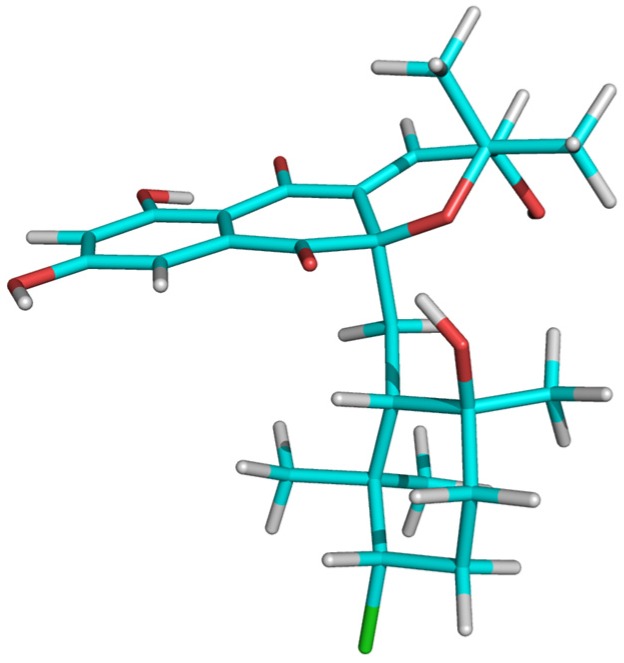
Energy-minimized molecular model of MDN-0170 (**1**) showing its relative stereochemistry.

Establishing the relative stereochemistry of the dihydropyran ring required an evaluation of the energy-minimized molecular models of the two possible epimers of compound **1** at C-3 for their compatibility with the experimental NMR data (observed nOes and coupling constant between H-3 and H-4). One of the epimers (epimer *a*) has the hydroxyl group with the same relative configuration as the chlorine atom at C-3 found in CNQ525.510A [5,6] while the other epimer (epimer *b*) has the opposite configuration for this hydroxyl group. Starting from the reported crystal structure of the related antibiotic A80915C [24] (Figure 5), the molecular models of the two possible epimers at C-3 were created and energy-minimized using Chem3D 12.0 Pro software (see Figure S8). The coupling constant between H-3 and H-4 has a value of 6.9 Hz (Figure 4) which perfectly agrees with the corresponding dihedral angle (23.1°) observed in the molecular model of epimer *b*, and it is not compatible with the dihedral angle (−97.5°) observed in the molecular model of epimer *a*. Interestingly, the corresponding coupling constant reported for the equivalent protons in CNQ525.510A, which has the relative configuration displayed by epimer *a*, has a value of 1.8 Hz [5] that would be compatible with the dihedral angle of −97.5° measured in the molecular model of epimer *a*. On the other hand, the distance measured between protons H-3 and H-4 equals 2.83 Å and 2.41 Å in the molecular models of epimers *a* and *b*, respectively. A very strong NOESY correlation is observed between these two protons in compound **1**, which would be more compatible with the distance measured in the model of epimer *b*, although the distance measured in the model of epimer *a* would likely result in a significant NOESY correlation. In fact, such a correlation is observed for the equivalent protons in CNQ525.510A (which has the relative configuration displayed by epimer *a*) [5]. The definite evidence confirming that the dihydropyran ring has the relative configuration displayed by epimer *b* came from the almost equally intensely strong NOESY correlations observed between H-3 and both the C-18 and C-19 geminal methyl groups (Figure 4). In the model of epimer *b*, the distances between H-3 and each of the geminal methyl carbons are 2.75 Å and 2.78 Å explaining the equally intense NOESY correlation of H-3 with both groups. However, in the model of epimer *a*, just one strong correlation would be observed since the distances between H-3 and each of the geminal methyl carbons are 2.77 Å and 3.54 Å (this methyl is antiperiplanar with respect to H-3). Thus, the dihydropyran ring in MDN-0170 (**1**) has the relative configuration displayed by epimer *b* (Figure 6). The relative configuration of H-3 in MDN-0170 is therefore opposite to that reported for CNQ525.510A [5]. 

It could be speculated that the precursor of MDN-0170 (**1**) might be the corresponding chlorinated compound at C-3 with the same configuration found for CNQ525.510A [5] and that an enzymatic hydroxylation via a nucleophilic substitution (with chloride ion as the leaving group) at this position, proceeding with inversion of configuration, would render the relative stereochemistry found for compound **1**. In fact, the presence of a hydroxyl substitution at C-3 on the dihydropyran ring has been previously reported in a napyradiomycin of the “C type” [8]. Not surprisingly, that napyradiomycin and MDN-0170 (**1**) share the same relative configuration for the chiral centers on the dihydropyran ring suggesting a common enzymatic mechanism for the incorporation of the hydroxyl group at C-3. MDN-0170 (**1**) represents the first napyradiomycin of the “B type” bearing a hydroxyl group rather than a chlorine at this position.

Finally, NMR also provided evidence of the stereochemical relationship between C-10a and C-12. Analysis of the coupling constants involving the geminal H-11 protons showed H-11a as a broad doublet (15.7 Hz and 1.4 Hz, the small coupling measured after resolution enhancement using a gaussian window function) and H-11b as a double doublet (15.7 Hz and 7.8 Hz). In each multiplet, the large 15.7 Hz coupling constant corresponds to the geminal coupling between the H-11 methylene protons while the small coupling is the vecinal coupling with H-12. These values indicate a dihedral angle between H-12 and H11a (*J* = 1.4 Hz) close to −74° while the dihedral angle between H-12 and H-11b (*J* = 7.8 Hz) would be close to 141°. Interestingly, in the energy-minimized model of MDN-0170 (**1**) the dihedral angles measured are −74.2° and 170° respectively, in good agreement with the observed coupling constants, bearing in mind that the torsion around the C-11–C-12 bond is more flexible than the rigid rings of the molecule. Regarding the NOESY correlations, the axial C-22 methyl group showed correlations with H-11a (medium) and H-11b (weak) while the equatorial C-21 methyl showed a strong correlation with H-11a but no correlation with H-11b. These key correlations are in agreement with the corresponding distances observed in the energy-minimized model of MDN-0170. On the other hand, although very weak, a clear genuine NOESY correlation is observed between the aromatic H-9 proton and the equatorial C-21 methyl group. This correlation is in agreement with the molecular model of MDN-0170 (**1**) in which the corresponding distance equals 3.7 Å and definitely validates the relative stereochemistry of the whole molecule, proving that the stereochemical relationship between C-10a and C-12, which relates the two independent stereoclusters (cyclohexane ring and dihydropyran ring), is the same as that found in CNQ525.510A [5,6]. As already mentioned, it is expected that the absolute configuration of all the chiral centers except C-3 in MDN-0170 (**1**) is the same as that reported for CNQ525.510A [5,6] since both compounds must share a common biosynthetic route [21]. 

### 2.4. Evaluation of Antimicrobial Activity

#### Antibacterial and Antifungal Activities

Compounds **1**–**4** were evaluated for their antibacterial and antifungal properties against a clinical isolate of MRSA, *E. coli*, *A. fumigatus*, and *C. albicans* (Table 2). Compounds **2** and **3** showed outstanding antibacterial activity inhibiting the growth of MRSA with minimum inhibitory concentration (MIC) values between 0.5 and 8 μg/mL, while compounds **1** and **4** did not inhibit the growth of MRSA when tested at 64 μg/mL. Otherwise, none of the compounds displayed activity against *E. coli*, *A. fumigatus*, and *C. albicans* when tested at 64 μg/mL. 

## 3. Materials and Methods

### 3.1. General Experimental Procedures

Optical rotation was measured with a Jasco P-2000 polarimeter (JASCO Corporation, Tokyo, Japan). Infrared spectra were measured with a JASCO FT/IR-4100 spectrometer (JASCO Corporation) equipped with a PIKE MIRacle™ single reflection ATR accessory. NMR spectra were recorded on a Bruker Avance III spectrometer (500 and 125 MHz for ^1^H and ^13^C NMR, respectively) equipped with a 1.7 mm TCI MicroCryoProbe™ (Bruker Biospin, Fällanden, Switzerland). Chemical shifts were reported in ppm using the signals of the residual solvents as internal reference (δ_H_ 3.31 and δ_C_ 49.0 for CD_3_OD). LC-UV-MS analysis was performed on an Agilent 1100 (Agilent Tehcnologies, Santa Clara, CA, USA) single quadrupole LC-MS system as previously described [25]. ESI-TOF and MS/MS spectra were acquired using a Bruker maXis QTOF (Bruker Daltonik GmbH, Bremen, Germany) mass spectrometer coupled to an Agilent 1200 LC (Agilent Technologies, Waldbronn, Germany). Acetone used for extraction was analytical grade. Solvents employed for isolation were HPLC grade. Molecular models were generated using Chem3D Pro 12.0 (CambridgeSoft, PerkinElmer Informatics, Waltham, MA, USA). The structures were energy-minimized by molecular mechanics with the MM2 force field using an RMS value of 0.001 as gradient convergence criteria. Molecular modelling figures were generated with PyMol (W. L. DeLano, The PyMOL Molecular Graphics System, DeLano Scientific LLC, Palo Alto, CA, USA, 2002).

### 3.2. Taxonomical Identification of the Producing Microorganism

Genomic DNA from CA-271078 was isolated employing the following protocol. Strain CA-271078 was grown on ATCC-2-M medium (soluble starch 20 g/L, glucose 10 g/L, NZ Amine Type E 5 g/L, meat extract 3 g/L, peptone 5 g/L, yeast extract 5 g/L, sea salts 30 g/L, calcium carbonate 1 g/L, pH 7) for about 96 h. The broth (1.5 mL) was centrifuged for 15 min at 13,000 rpm and 4 °C in an eppendorf tube. The supernatant was discarded and the pellet was re-suspended in 800 μL of extraction buffer (0.2% SDS, 50 mM EDTA, pH 8.5) and heated at 70 °C for 30 min. The resulting mixture was then centrifuged for 15 min at 13,000 rpm and 4 °C. The supernatant was then transferred to an eppendorf tube containing 60 μL of sodium acetate (3 M, pH 5.2). The mixture was incubated at 4 °C for 2 h and then centrifuged for 15 min at 13,000 rpm and 4 °C. Five hundred microliters of the supernatant were transferred to an eppendorf tube containing 1 mL of ^i^ PrOH and the mixture was incubated at 4 °C overnight. The next day the content was centrifuged (15 min, 13,000 rpm, 4 °C), the supernatant was discarded, and the pellet was washed with 200 μL of 70% ethanol. The washed pellet was centrifuged in the same conditions (15 min, 13,000 rpm, 4 °C), the supernatant was discarded, and the pellet was dried for several hours at room temperature. The pellet (genomic DNA) was then resuspended in 100 μL of sterile water.

The 16S rRNA gene was PCR-amplified employing the universal eubacterial primers fD1 (5′-AGAGTTTGATCCTGGCTCAG-3′) and rP2 (5′-ACGGCTACCTTGTTACGACTT-3′). PCR mixtures contained 5 μL of PCR buffer (10×), 4 μL of dNTPs (2.5 mM each), 0.5 μL of each of the primers (100 μM), 2 μL of a 1/50 dilution of the genomic DNA, and 0.4 μL of Taq Polymerase (5 U/μL) in a total volume of 50 μL. The PCR product was purified and sequenced at Secugen S. L. (Madrid, Spain) employing the above primers and the internal primers 926F (5′-AAACTYAAAKGAATTGACGG-3′) and 1100R (5′-GGGTTGCGCTCGTTG-3′). The resulting DNA sequence lectures were aligned and visually inspected with Bionumerics 6.6 to obtain a nearly complete (1359 nt) sequence (Text S9).

### 3.3. Fermentation of the Producing Microorganism

A 1 L fermentation of strain CA-271078 was generated as follows: a seed culture of the strain was obtained by inoculating two 25 × 150 mm tubes containing 16 mL of ATCC-2-M medium (soluble starch 20 g/L, glucose 10 g/L, NZ Amine Type E 5 g/L, meat extract 3 g/L, peptone 5 g/L, yeast extract 5 g/L, sea salts 30 g/L, calcium carbonate 1 g/L, pH 7) with 0.8 mL of a freshly thawed inoculum stock of the producing strain. The tubes were incubated in a rotary shaker at 28 °C, 70% relative humidity, and 220 rpm for about 96 h. The fresh inoculum thus generated was mixed and employed to inoculate twenty 250 mL flasks, each containing 50 mL of R358 medium (2.5% v/v) (soluble starch 10 g/L, yeast extract 4 g/L, peptone 2 g/L, KBr stock solution 5 mL/L (stock containing 8 g/L), FeSO_4_·7H_2_O stock solution 5 mL/L (stock containing 8 g/L), sea salts 30 g/L, pH adjusted to 7.0). The inoculated flasks were incubated in a rotary shaker at 28 °C, 70% relative humidity, and 220 rpm for six days before harvesting.

### 3.4. Extraction and Bioassay Guided Isolation

The fermentation broth (1 L) was extracted with acetone (1 L) under continuous shaking at 220 rpm for 2 h. The mycelium was separated by filtration and the supernatant (ca. 2L) was concentrated to 1 L under reduced pressure. The aqueous crude extract was extracted with ethyl acetate to afford an ethyl acetate extract (0.226 g). After confirming bioactivity against MRSA, this ethyl acetate extract was chromatographed on Sephadex LH-20 (30 × 500 mm) using methanol/dichloromethane (2:1) to afford 24 fractions of 15 mL. They were combined into ten fractions according to LC-UV-MS chemical profiles: fractions A–J. Fractions C (0.029 g), D (0.030 g), F (0.060 g), G (0.010 g), and H (0.058 g) were the most active against MRSA.

Fraction F (0.060 g) was chromatographed on Sephadex LH-20 using mixtures of chloroform/methanol (2:1) affording five fractions grouped according to LC-UV-MS profiles. The fifth fraction was purified by reverse-phase semipreparative HPLC (column Agilent Zorbax RX-C8, 9.4 × 250 mm, 7 μm; 3 mL/min, UV detection at 210 and 260 nm) with a linear gradient of CH_3_CN/H_2_O with 0.1% trifluoroacetic acid, from 60% to 75% CH_3_CN over 50 min yielding **1** (0.3 mg, *t*_R_ 30 min).

Fraction G (0.015 g) was subjected to reversed-phase semipreparative HPLC (column Agilent Zorbax RX-C8, 9.4 × 250 mm, 7 μm; 3 mL/min, UV detection at 210 and 260 nm) with a linear gradient of CH_3_CN/ H_2_O with 0.1% trifluoroacetic acid, from 50% to 80% CH_3_CN over 50 min, yielding **1** (0.5 mg, *t*_R_ 31 min).

Fraction H (0.058 g) was chromatographed on Sephadex LH-20 (30 × 250 mm) using mixtures of chloroform/methanol (2:1), affording 16 fractions of 5 mL. They were reduced to four final fractions according to LC-UV-MS chemical profiles. Fraction 2 was subjected to reversed-phase semipreparative HPLC (column Agilent Zorbax RX-C8, 9.4 × 250 mm, 7 μm; 3 mL/min, UV detection at 210 and 260 nm) with a linear gradient of CH_3_CN/H_2_O with 0.1% trifluoroacetic acid, from 50% to 80% CH_3_CN over 50 min, yielding **2** (1.2 mg, *t*_R_ 40 min) and **3** (1.5 mg, *t*_R_ 42 min). Fraction 4 was subjected to reversed-phase semipreparative HPLC (column Agilent Zorbax RX-C8, 9.4 × 250 mm, 7 μm; 3 mL/min, UV detection at 210 and 260 nm) with a linear gradient of CH_3_CN/ H_2_O with 0.1% trifluoroacetic acid, from 50% to 80% CH_3_CN over 50 min, yielding **4** (2.2 mg, *t*_R_ 32 min).

MDN-0170 (**1**): yellow oil; [α]${}_{D}^{25}$ +6.3° (*c* 0.04, MeOH); IR (ATR) cm^−1^: 3350, 2955, 1712, 1680, 1618, 1265, 1082, 1017, 877, 779; (+)-ESI-TOFMS *m/z* 496.2080 [M + NH_4_]^+^ (calcd. for C_25_H_35_^35^ClNO_7_^+^, 496.2097), 479.1818 [M + H]^+^ (calcd. for C_25_H_32_^35^ClO_7_^+^, 479.1831), 461.1721 [M + H − H_2_O]^+^ (calcd. for C_25_H_30_^35^ClO_6_^+^, 461.1725), 443.1614 [M + H − 2H_2_O]^+^ (calcd. for C_25_H_28_^35^ClO_5_^+^, 443.1620); ^1^H and ^13^C NMR data see Table 1.

### 3.5. Antibacterial and Antifungal Assays

Compounds **1**–**4** were tested for their ability to inhibit the growth of Gram negative and Gram positive bacteria (*E. coli* MB2884 and methicillin-resistant *S. aureus* MRSA, MB5393), fungi (*A. fumigatus* ATCC46645), and yeast (*C. albicans* MY1055) following previously described methodologies [26,27,28]. Briefly, each compound was serially diluted in DMSO with a dilution factor of two to provide ten concentrations starting at 64 μg/mL for all the assays. The MIC was defined as the lowest concentration of compound that inhibited ≥95% of the growth of a microorganism after overnight incubation. The Genedata Screener software (Genedata, Inc., Basel, Switzerland) was used to process and analyze the data and also to calculate the RZ’ factor, which predicts the robustness of an assay [29]. In all experiments performed in this work, the RZ’ factor obtained was between 0.87 and 0.98.

## 4. Conclusions

Compounds **1**–**4** have been identified from the culture of *Streptomyces* sp. strain CA-271078, a *Streptomyces* strain related to *Streptomyces aculeolatus* NBRC 14824(T) which was isolated from an ascidian collected at the seaside in Baía Ana Chaves, Sao Tome (Sao Tome and Principe). Compounds **2** and **3** were able to inhibit strongly the growth of MRSA, one of the most common causes of hospital-acquired infections, with MIC values in the micromolar range. The activity found for compounds **2** and **3** against a clinical isolate of MRSA expands the panel of activity previously reported for these compounds against methicillin-sensitive *S. aureus* [12] to methicillin-resistant strains. Additionally, in view of the bioactivity displayed by some members of the family against Gram-positive bacteria and cancer cell lines, some conclusions about structure-activity relationships of these naphtoquinones can be established. A prenyl chain attached to C-10a or at least a double bond at position Δ^13,20^ seem to be essential for the biological activity of this structural class [4,5,6,12]. Using molecular modelling in combination with nOe and coupling constants analyses, the relative configuration of compound **1,** containing two independent stereoclusters, has been established. Biosynthetic arguments for the compound also allowed us to propose its absolute stereochemistry. This article constitutes the first report on the chemical composition of extracts from a marine derived *Streptomyces* strain related to *S. aculeolatus*, and has identified this strain as a new source of milbemycins and NPDs related compounds. The research described herein also confirms that marine derived actinomycetes continue to be a rich and underexploited source of new small molecules that could lead to the discovery of new antibiotics.

## Figures and Tables

**Table 1:** ^1^H and ^13^C NMR (500 and 125 MHz in CD_3_OD) data for compound **1**.

Position	δ ^1^H (mult, *J*, Hz)	δ ^13^C	Position	δ ^1^H (mult, *J*, Hz)	δ ^13^C
2	----	78.8, s	11	1.96, dd, 15.7, 1.4 2.22, dd, 15.7, 7.8	40.9, t
3	3.88, d, 6.9	67.0, d	12	1.55, d, 7.8	52.6, d
4	7.10, d, 6.9	134.2, d	13	----	72.1, s
4a	----	140.2, s	14	1.55, m 1.77, m	42.2, t
5	----	190.6, s	15	1.82, m 1.91, dd, 13.7, 4.0	31.6, t
5a	----	111.7, s	16	3.70, dd, 12.2, 4.0	72.2, d
6	----	166.3, s	17	----	41.8, s
7	6.60, d, 2.0	109.7, d	18	1.04, s	25.6, q
8	----	167.3, s	19	1.44, s	24.7, q
9	6.96, d, 2.0	109.5, d	20	1.17, s	24.4, q
9a	----	137.8, s	21	0.56, s	29.2, q
10	----	196.3, s	22	0.71, s	16.3, q
10a	----	83.4, s			

**Table 2:** Antimicrobial activity (μg/mL) of compounds **1**–**4**.

MIC (μg/mL)								
Microbial Strain	Strain Number	(1)	(2)	(3)	(4)	V	R	A
MRSA	MB5393	>64	4–8	0.5–1	>64	2–4		
*E. coli*	MB2884	>64	>64	>64	>64		6.5–12.5	
*A. fumigatus*	ATCC46645	>64	>64	>64	>64			4
*C. albicans*	MY1055	>64	>64	>64	>64			2–4

V (vancomycin), R (rifampicin), A (amphotericin B).

## Supplementary Materials

The following are available online at www.mdpi.com/1660-3397/14/10/188/s1, Figure S1: Electrospray-time of flight (ESI-TOF) (A) and UV (B) spectra for compound **1**; Figure S2: ^1^H NMR spectrum (CD_3_OD, 500 MHz) of compound **1**; Figure S3: ^13^C NMR spectrum (CD_3_OD, 125 MHz) of compound **1**; Figure S4: COSY spectrum of compound **1**; Figure S5: HSQC spectrum of compound **1**; Figure S6: HMBC spectrum of compound **1**; Figure S7: NOESY spectrum of compound **1**; Figure S8: Energy-minimized models of the two possible epimers at C-3 of compound **1**; Text S9: 16S rRNA gene sequence from CA-271078.
