# Supplementary material for: MDN-0170, a New Napyradiomycin from Streptomyces sp. Strain CA-271078
Source: Mar Drugs. 2016 Oct 18;14(10):188. doi: 10.3390/md14100188 (PMC5082336; doi:10.3390/md14100188)
Supplement: Supplementary file 1 [file marinedrugs-14-00188-s001.pdf]

Supplementary Materials: MDN-0170, a New Napyradiomycin from *Streptomyces* sp. Strain CA-271078

Rodney Lacret, Ignacio Pérez-Victoria, Daniel Oves-Costales, Mercedes de la Cruz, Elizabeth Domingo, Jesús Martín, Caridad Díaz, Francisca Vicente, Olga Genilloud and Fernando Reyes

List of Supplementary Materials:

Figure S1. Electrospray-time of flight (ESI-TOF) (**A**) and UV (**B**) spectra for compound **1**.
Figure S2. ^{1}H NMR spectrum (CD_3OD, 500 MHz) of compound **1**.
Figure S3. ^{13}C NMR spectrum (CD_3OD, 125 MHz) of compound **1**.
Figure S4. COSY spectrum of compound **1**.
Figure S5. Heteronuclear single quantum coherence (HSQC) spectrum of compound **1**.
Figure S6. HMBC spectrum of compound **1**.
Figure S7. NOESY spectrum of compound **1**.
Figure S8. Energy-minimized molecular models of the two possible epimers at C-3 of compound **1**
Text S9. 16S rRNA gene sequence from strain CA-271078

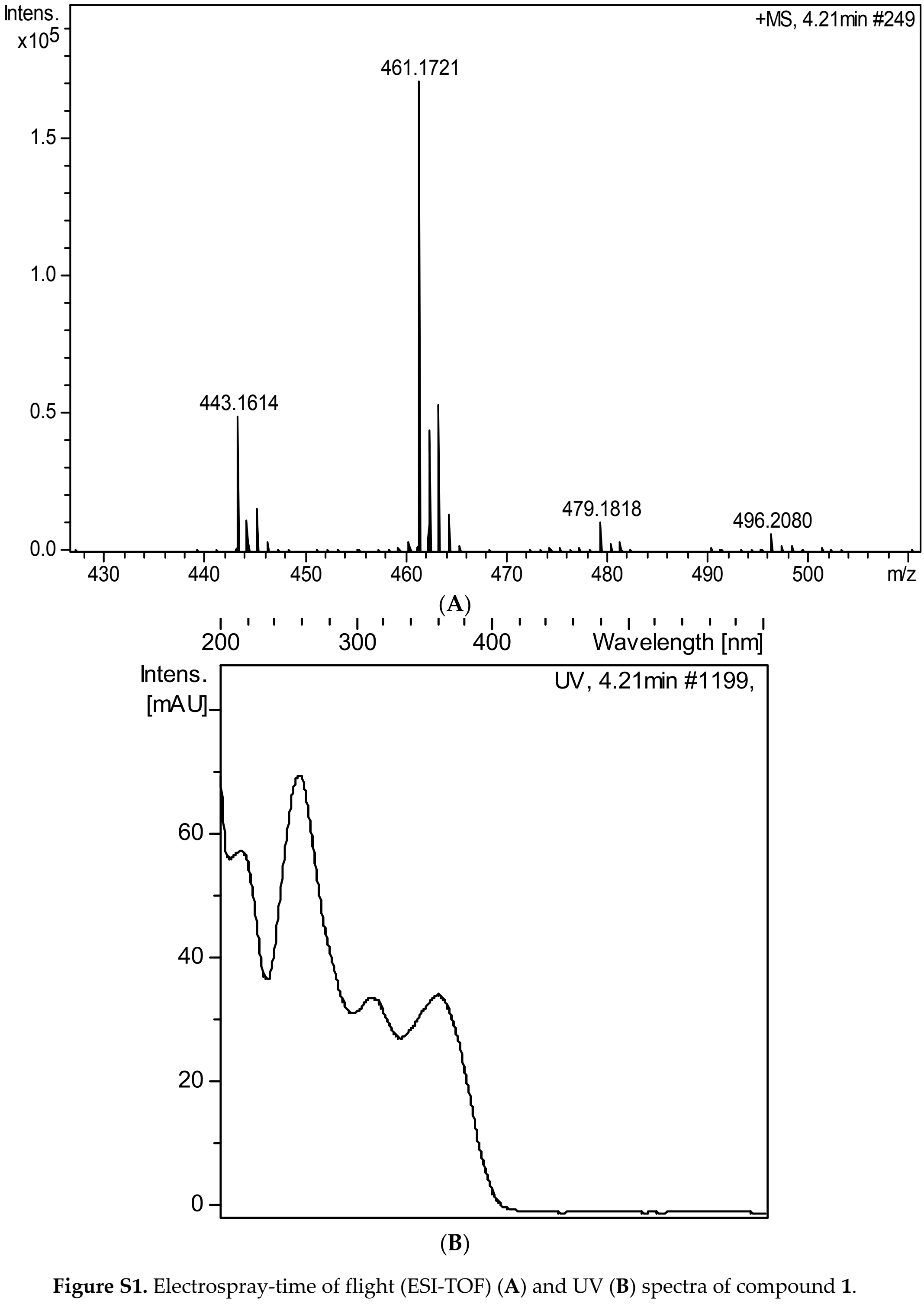

(**B**)

Figure S1. Electrospray-time of flight (ESI-TOF) (**A**) and UV (**B**) spectra of compound **1**.

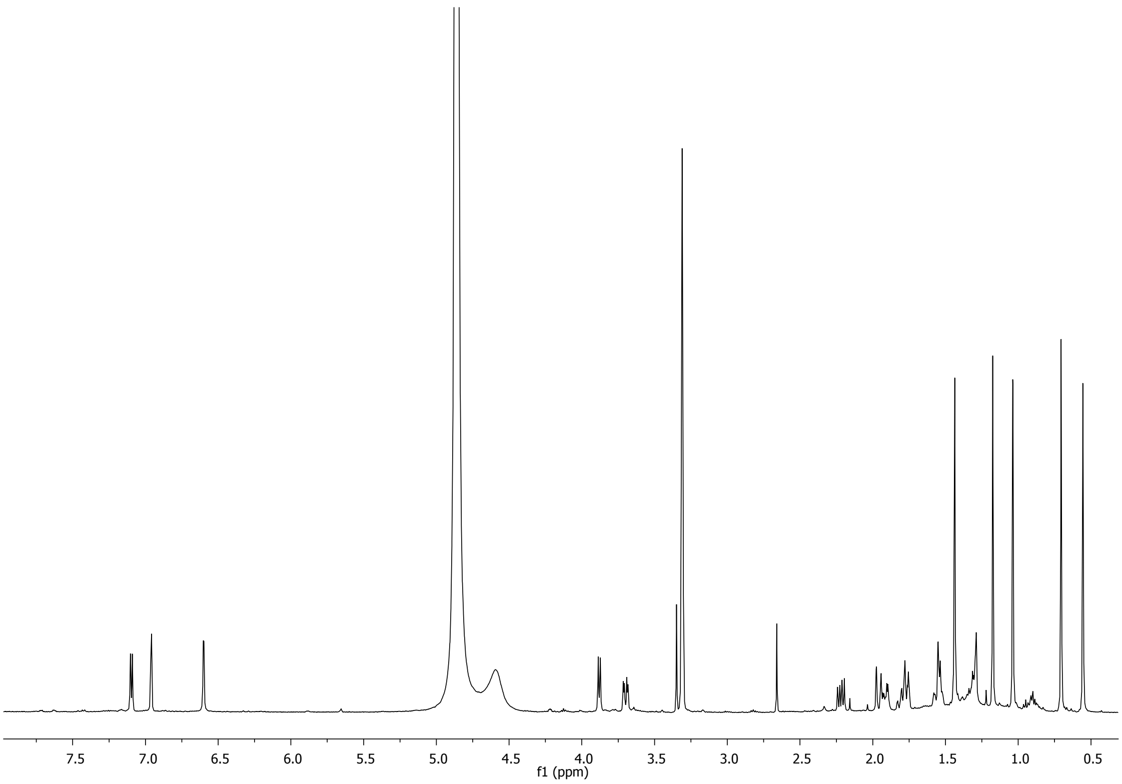

Figure S2. ^{1}H NMR (CD_3OD, 500 MHz) of compound **1**.

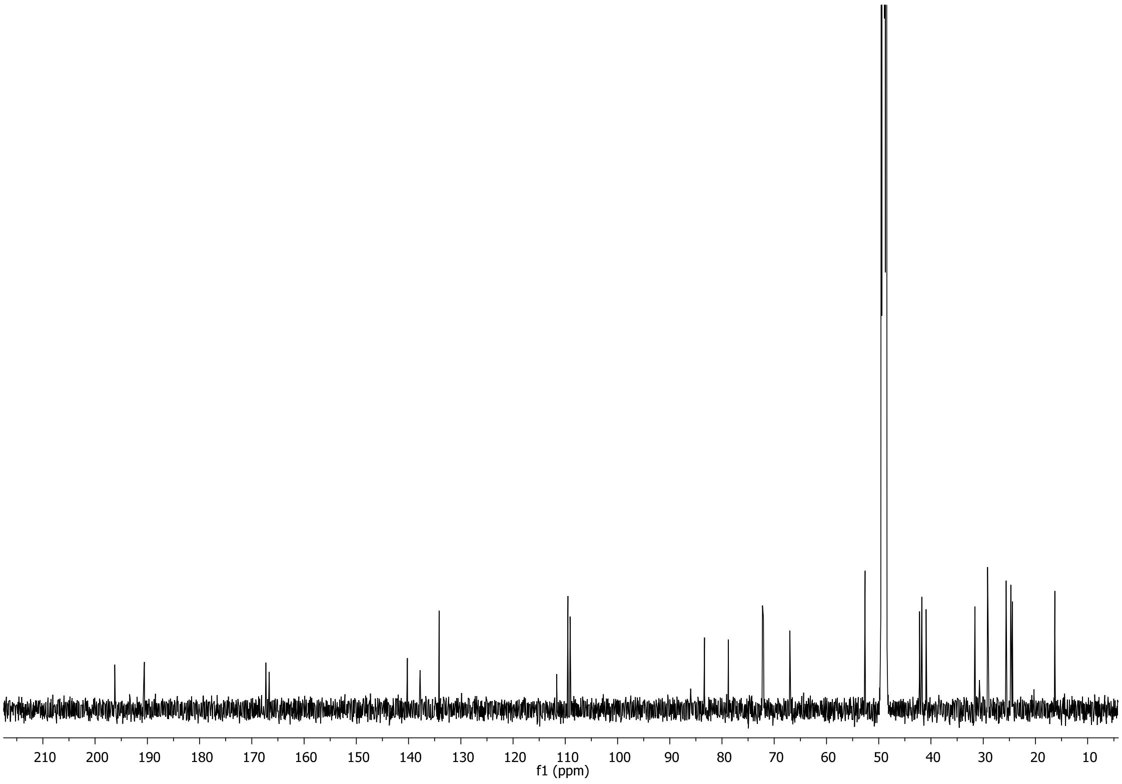

Figure S3. ^{13}C NMR (CD_3OD, 125 MHz) of compound **1**.

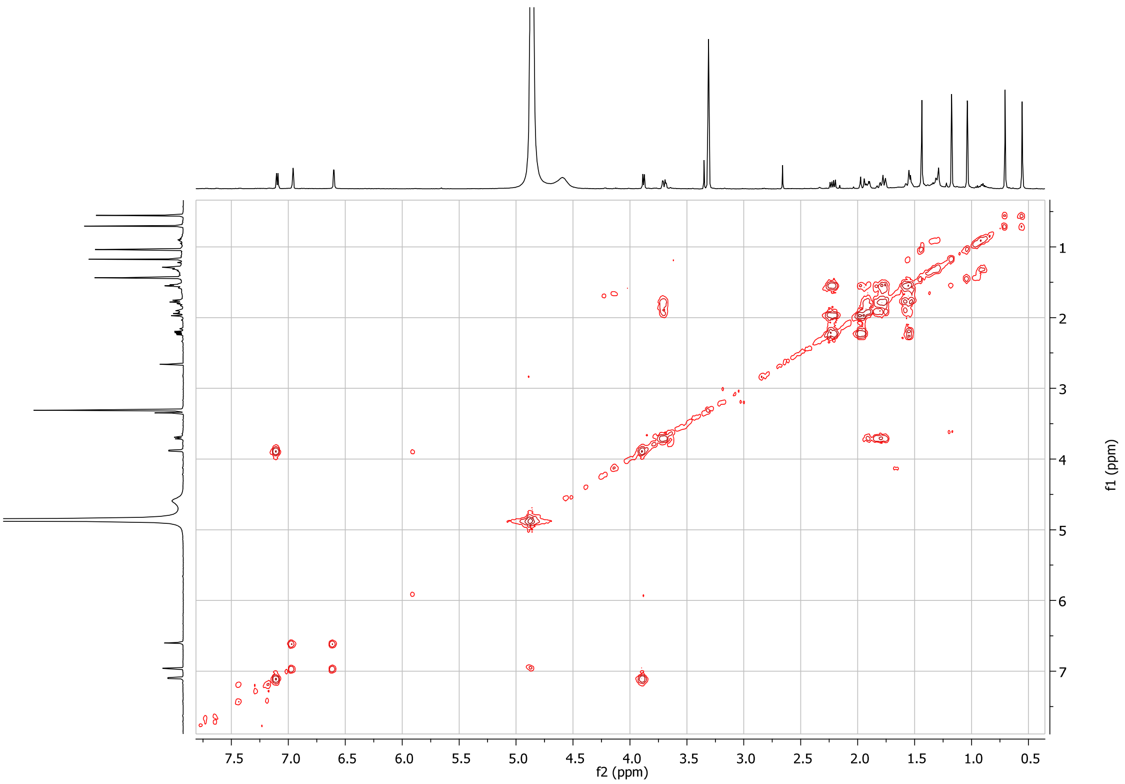

Figure S4. COSY of compound **1**.

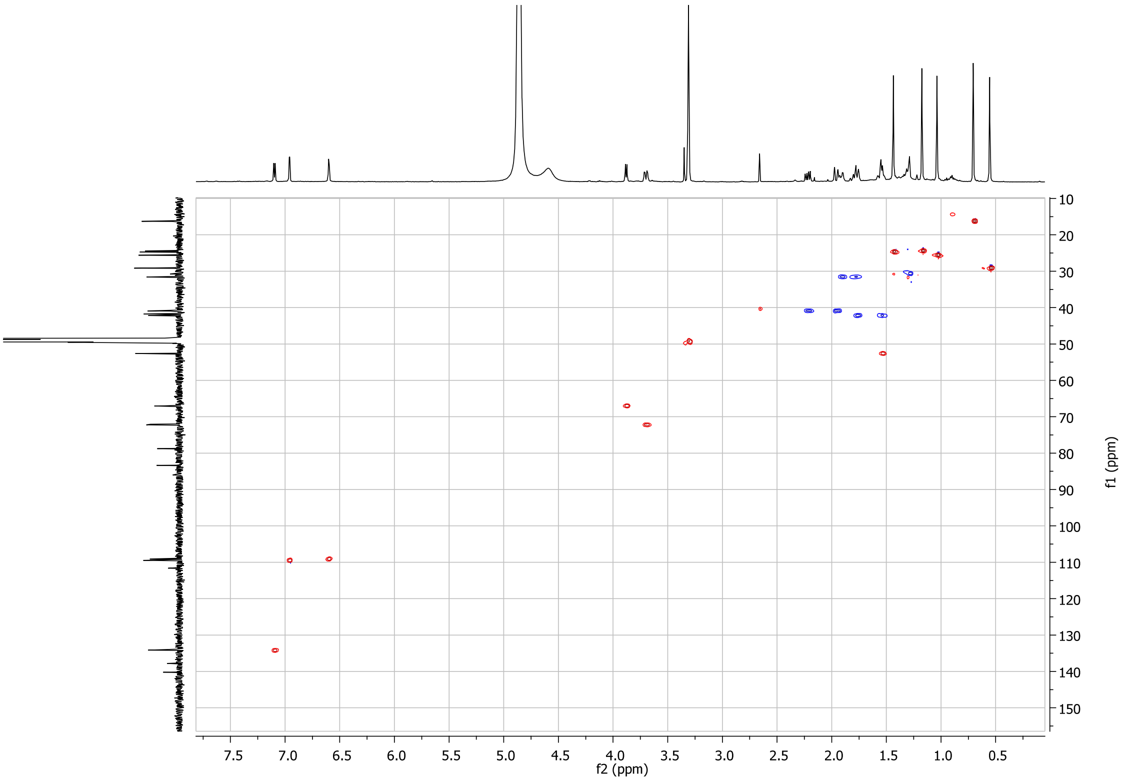

Figure S5. Heteronuclear single quantum coherence (HSQC) spectrum of compound **1**.

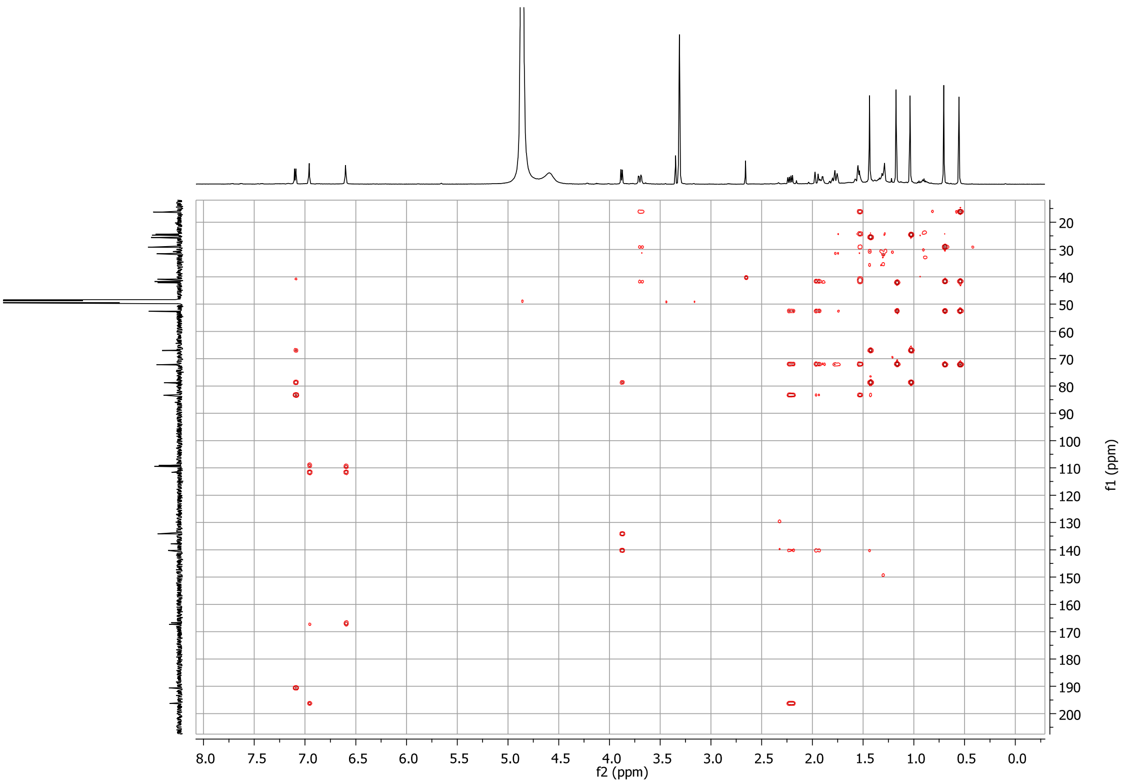

Figure S6. HMBC of compound **1**.

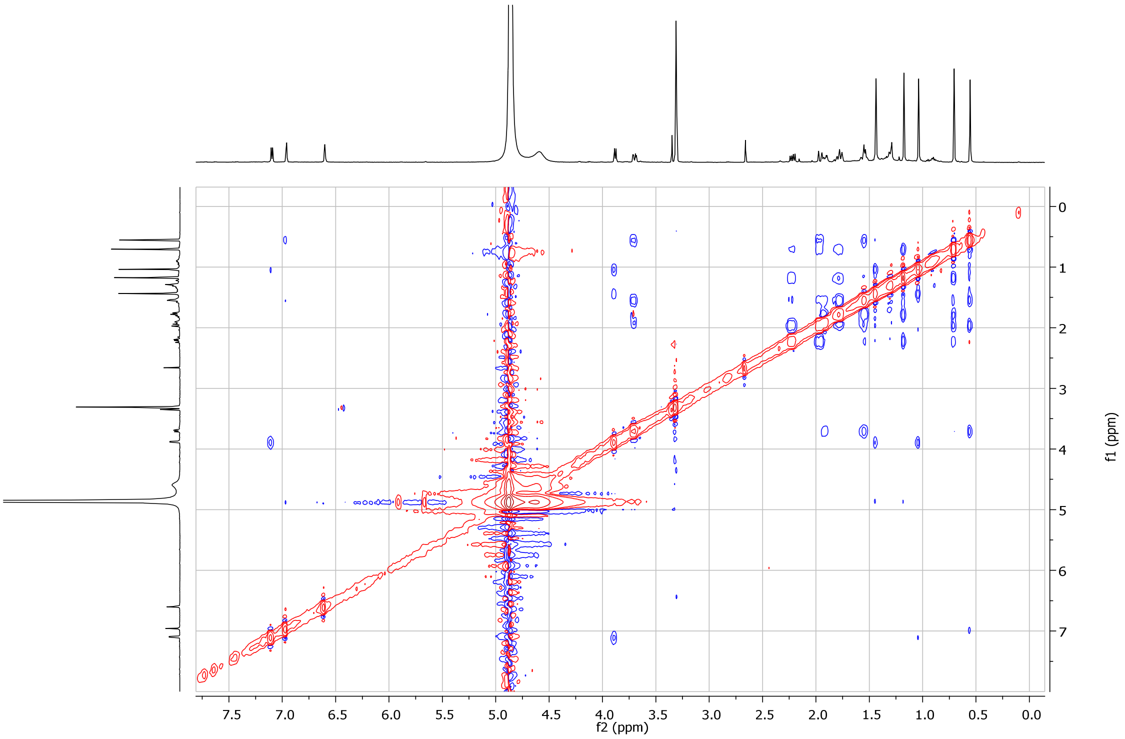

Figure S7. NOESY of compound **1**.

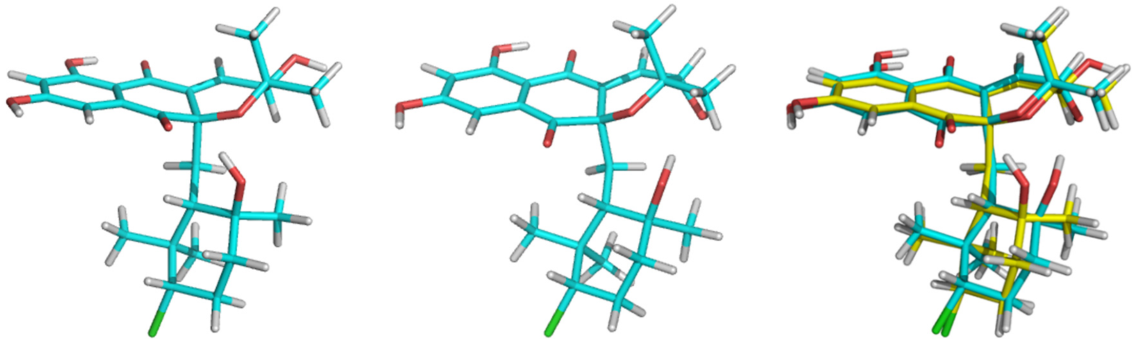

Epimer **a** Epimer **b** Overlay: **a** (lemon) **b** (cyan)

Figure S8. Energy-minimized molecular models of the two possible epimers at C-3 of compound **1.**

>CA-271078 16S rRNA gene sequence
ATGATCCGGTTTCGGCCGGGGATTAGTGGCGAACGGGTGAGTAACACGTGGGCAA
TCTGCCCTGCACTCTGGGACAAGCCCTGGAAACGGGGTCTAATACCGGATAGTAC
CTTCGGGCGCATGCCTGTTGGTGGAAAGCTCCGGCGGTGCAGGATGGGCCCGCGG
CCTATCAGCTTGTTGGTGGGGTGATGGCCTACCAAGGCGACGACGGGTAGCCGGC
CTGAGAGGGCGACCGGCCACACTGGGACTGAGACACGGCCCAGACTCCTACGGG
AGGCAGCAGTGGGGAATATTGCACAATGGGCGCAAGCCTGATGCAGCGACGCCG
CGTGAGGGATGACGGCCTTCGGGTTGTAAACCTCTTTCAGCAGGGAAGAAGCGAG
AGTGACGGTACCTGCAGAAGAAGCGCCGGCTAACTACGTGCCAGCAGCCGCGGT
AATACGTAGGGCGCAAGCGTTGTCCGGAATTATTGGGCGTAAAGAGCTCGTAGGC
GGCTTGTCGCGTCGGATGTGAAAGCCCGGGGCTTAACCCCGGGTCTGCATTCGATA
CGGGCAGGCTGGAGTTCGGTAGGGGAGATCGGAATTCCTGGTGTAGCGGTGGAAT
GCGCAGATATCAGGAGGAACACCGGTGGCGAAGGCGGATCTCTGGGCCGATACT
GACGCTGAGGAGCGAAAGCGTGGGGAGCAAACAGGATTAGATACCCTGGTAGTC
CACGCCGTAAACGGTGGGCACTAGGTGTGGGCGGCATTCCACGTCGTCCGTGCCG
TAGCTAACGCATTAAGTGCCCCGCCTGGGGAGTACGGCCGCAAGGCTAAAACTCA
AAGGAATTGACGGGGGCCCGCACAAGCGGCGGAGCATGTGGCTTAATTCGACGC
AACGCGAAGAACCTTACCAAGGCTTGACATACACCGGAAACACCTGGAGACAGG
TGCCCCCTTGTGGTCGGTGTACAGGTGGTGCATGGCTGTCGTCAGCTCGTGTCGTG
AGATGTTGGGTTAAGTCCCGCAACGAGCGCAACCCTTGTCCTGTGTTGCCAGCACG
CCTTTCGGGGTGGTGGGGACTCACGGGAGACTGCCGGGGTCAACTCGGAGGAAGG
TGGGGACGACGTCAAGTCATCATGCCCCTTATGTCTTGGGCTGCACACGTGCTACA
ATGGCCGGTACAATGAGCTGCGATACCGCGAGGTGGAGCGAATCTCAAAAAGCC
GGTCTCAGTTCGGATTGGGGTCTGCAACTCGACCCCATGAAGTCGGAGTCGCTAGT
AATCGCAGATCAGCATTGCTGCGGTGAATACGTTCCCGGGCCTTGTACACACCGCC
CGTCACGTCACGAAAGTCGGTAACACCCGAAGCCGGTGGCC

Text S9. 16S rRNA gene sequence from strain CA-271078.
